# Fine scale spatial investigation of multiple insecticide resistance and underlying target-site and metabolic mechanisms in *Anopheles gambiae* in central Côte d’Ivoire

**DOI:** 10.1038/s41598-020-71933-8

**Published:** 2020-09-15

**Authors:** Welbeck A. Oumbouke, Patricia Pignatelli, Antoine M. G. Barreaux, Innocent Z. Tia, Alphonsine A. Koffi, Ludovic P. Ahoua Alou, Eleanore D. Sternberg, Matthew B. Thomas, David Weetman, Raphael N’Guessan

**Affiliations:** 1 Department of Disease Control, London School of Hygiene and Tropical Medicine, London, UK; 2 Institut Pierre Richet (IPR), Institut National de Santé Publique (INSP), Bouaké, Côte d’Ivoire; 3 Department of Vector Biology, Liverpool School of Tropical Medicine, Pembroke Place, Liverpool, UK; 4 School of Biological Sciences, University of Bristol, Bristol, UK; 5 Department of Entomology, Center for Infectious Disease Dynamics, The Pennsylvania State University, University Park, PA 16802 USA

**Keywords:** Gene expression analysis, Microarray analysis, Genetics

## Abstract

Routine monitoring of occurrence, levels and mechanisms of insecticide resistance informs effective management strategies, and should be used to assess the effect of new tools on resistance. As part of a cluster randomised controlled trial evaluating a novel insecticide-based intervention in central Côte d’Ivoire, we assessed resistance and its underlying mechanisms in *Anopheles gambiae* populations from a subset of trial villages. Resistance to multiple insecticides in *An. gambiae s.s.* and *An. coluzzii* was detected across villages, with dose–response assays demonstrating extremely high resistance intensity to the pyrethroid deltamethrin (> 1,500-fold), and mortality following exposure to pyrethroid-treated bednets was low (< 30% mortality in cone bioassays). The 1014F *kdr* mutation was almost fixed (≥ 90%) in all villages but the 1575Y *kdr*-amplifying mutation was relatively rare (< 15%). The carbamate and organophosphate resistance-associated *Ace-1* G119S mutation was also detected at moderate frequencies (22–43%). Transcriptome analysis identified overexpression of P450 genes known to confer pyrethroid resistance (*Cyp9K1*, *Cyp6P3*, and *Cyp6M2*), and also a carboxylesterase (*COEAE1F*) as major candidates. *Cyp6P3* expression was high but variable (up to 33-fold) and correlated positively with deltamethrin resistance intensity across villages (r^2^ = 0.78, P = 0.02). Tools and strategies to mitigate the extreme and multiple resistance provided by these mechanisms are required in this area to avoid future control failures.

## Introduction

Insecticide-based control methods continue to play a crucial role in reducing vector-borne diseases. Insecticides are deployed against malaria mosquitoes most commonly via long lasting insecticidal nets (LLINs) and indoor residual spraying (IRS). The significant increase in coverage with LLINs over the past 20 years has been associated with a marked reduction in malaria burden^1^. However, recent estimates suggest that progress has stalled, with insecticide resistance likely one of the major contributing factors. Whilst selection from other sources, especially agriculture^2^, may be important in some areas, there is evidence that the wide scale use of IRS and particularly LLINs is contributing to selection for pyrethroid resistance in major African vectors of malaria^3^. Resistance to pyrethroids is likely to increase further over the coming years, given that pyrethroids remain an important component of all currently available bednets, including newer dual-action LLINs^4–6^.

Until recently, only four classes of insecticides (pyrethroids, organochlorines, carbamates and organophosphates) were licenced for use to control adult mosquito vectors. The pyrrole insecticide, chlorfenapyr, and the neonicotinoid, clothianidin, have recently been added to this list and are deployed either alone (for IRS) or in combination with pyrethroids (for LLINs)^7–9^. Except for these new insecticide classes, resistance to all currently available insecticides has been documented in *Anopheles* mosquito species across much of sub-Saharan Africa^10–14^. The best known mechanisms conferring resistance to insecticides are target site modification and increased detoxification. Substitutions in the para voltage-gated sodium channel (VSGC)—the target site for pyrethroids and DDT^15–17^ (L1014F and L1014S)—are widespread in *An. gambiae* and confer knock down resistance (*kdr*), with a third variant (N1575Y)^17^ capable of amplifying resistance where present^18^. A further mutation (G119S) in acetylcholinesterase (*Ace-1*) causes resistance to organophosphate and carbamate insecticides, which target this enzyme^19–21^. The G119S mutation is associated with a fitness cost in the absence of insecticides^22^ but *Ace-1* gene duplication, coupling resistant and susceptible alleles, or multiple resistant alleles on the same chromosome, has emerged in *An. gambiae* mosquitoes to offset deleterious effects^23^.

Metabolic resistance arises from enhanced detoxification of insecticides. Three classes of metabolic enzymes, carboxylesterases (COEs), glutathione-S-transferases (GSTs) and cytochrome P450s have been linked with resistance in various species of mosquitoes, with the latter most frequently implicated in metabolism of pyrethroids and carbamates^10,24–26^. Overexpression of several P450s has been associated with insecticide resistance, but relatively few have been validated as metabolizers in vitro, and thus only these can be regarded definitively as candidates capable of causing resistance. Notably, CYP6M2, CYP6P3 and CYP9K1 have all been validated not only as pyrethroid-metabolizers but also of unrelated insecticides (DDT, bendiocarb and pyriproxyfen, respectively) demonstrating how the substrate flexibility of some P450s can cause cross-resistance by metabolizing insecticides from diverse classes^10,27,28^.

Here we report on a study aimed at evaluating the current status of insecticide resistance in malaria vectors in central Côte d’Ivoire. Previous research has shown that *Anopheles* malaria vectors in Côte d’Ivoire have developed resistance to all of the four traditional classes of approved adulticides^21,29,30^. Resistance mechanisms detected in Côte d’Ivoire to date include *kdr* and *Ace-1* (mutation and duplication)^29^ and, in *An. coluzzii* from the southern part of the country, overexpression of P450 genes, especially *Cyp6M2* and *Cyp6P3*^10^. However, information on resistance intensity and a comprehensive assessment of the genetic mechanisms driving resistance in *An. gambiae* is lacking (and especially for central Côte d’Ivoire). The present study was thus conducted prior to the onset of a cluster randomized controlled trial (CRT) of the In2Care EaveTubes^31^, to characterize insecticide resistance across a subset of villages and provide a baseline against which future changes may be measured through the course of the CRT.

## Results

### Insecticide resistance and LLIN efficacy

**Figure 1:**
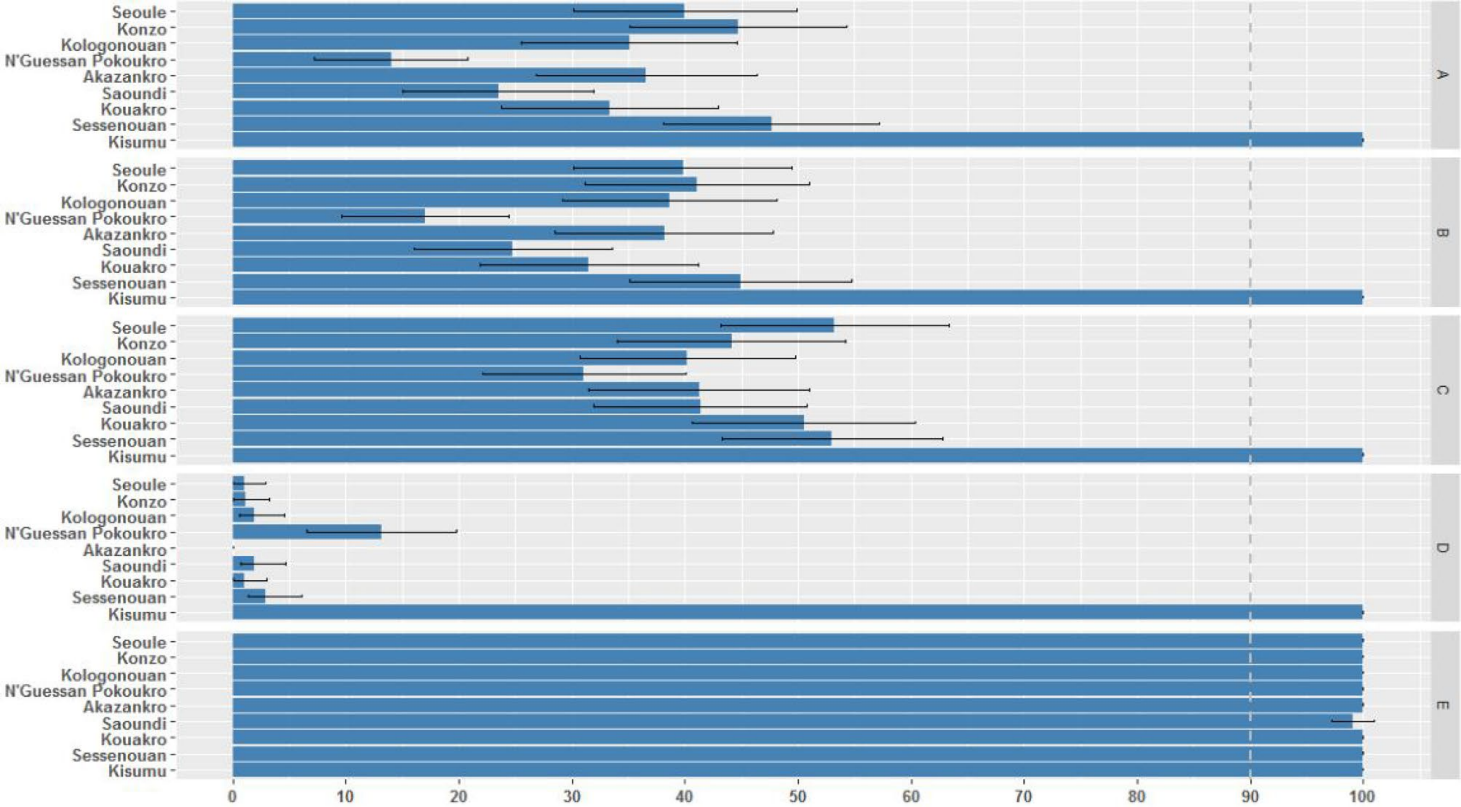
Twenty-four-hour percentage mortality of *An. gambiae* s.l. from each village exposed in diagnostic bioassays to (**A**) 0.05% deltamethrin, (**B**) 0.15% cyfluthrin, (**C**) 0.1% bendiocarb, (**D**) 4% DDT and (**E**) 1% pirimiphos methyl. Error bars represent 95% confidence intervals (Cis) and the dotted line indicates WHO resistance threshold.

Mortality rates of *An. gambiae* s.l. exposed to WHO diagnostic doses of deltamethrin, cyfluthrin, and bendiocarb were generally quite low with most villages below 50% (Fig. 1), and lower still for DDT (< 15%). Mortality results for the two pyrethroids were strongly correlated across villages (Spearman’s ρ = 0.98, n = 8, P < 0.001), and each was also positively correlated with bendiocarb mortalities, though neither significantly (maximum ρ = 0.64, minimum P = 0.09). There was significant variation among villages in bioassay mortalities for each insecticide, though there was no difference between groups of villages comprising the study arms for any insecticide (Table 1). For pirimiphos methyl, there was only one survivor out of over 800 females tested. However, the 1% dose used is four times the standard recommended diagnostic concentration, and results are best interpreted as evidence that higher intensity resistance is absent, rather than the population being fully susceptible.

**Table 1:** Generalised linear model testing the effects of village on bioassay mortality for each insecticide.

	Village (arm)		
	Wald χ^2^	df	P
Deltamethrin	35.245	6	0.000004
Cyfluthrin	25.53	6	0.0003
Bendiocarb	14.52	6	0.024
DDT	18.03	6	0.006
Pirimiphos methyl	Not calculated because mortality near 100%		

The intensity of resistance to deltamethrin measured using adapted CDC bottle assays was extremely high in all villages (RR_50_ range 1441 to 2405) (Table 2 and Table S1A&B). There was no difference between villages (overlapping 95% confidence intervals of LD 50 values in Table 2).

**Table 2:** Intensity of resistance to deltamethrin in *An. gambiae* s.l. from different villages in the study area prior to the study.

Strain	Slope (SE)	LD 50 (95% CI)	LD 95 (95% CI)	RR50
Kisumu^a^	1.3 (0.18)	0.015 (0.009–0.022)	0.261 (0.136–0.767)	–
Akanzakro	1.7 (0.2)	27.2 (20.3–35.2)	250.1 (166.7–451.0)	1873
Kologonouan	1.5 (0.1)	21.9 (15.8–28.5)	289.3 (190.0–534.4)	1504
Konzo	1.6 (0.1)	23.5 (19.1–28.3)	237.4 (173.7–358.2)	1617
Kouakro	1.7 (0.17)	22.4 (17.3–28.0)	213.5 (145.0–376.6)	1542
N’Guessan Pokoukro	2.1 (0.2)	33.7 (25.7–43.2)	207.0 (139.6–377.6)	2314
Saoundi	1.7 (0.1)	35.0 (28.9–41.9)	322.4 (237.8–477.3)	2405
Seoule	1.7 (0.1)	21.0 (17.2–25.0)	183.0 (139.1–261.3)	1441
Sessenouan	1.4 (0.1)	27.4 (20.8–34.8)	390.3 (256.3–708.0)	1883

*LD* lethal doses expressed in μg/mL; *RR50* resistance ratio, calculated by dividing the LD50 of the field mosquito population by that of the susceptible reference strain.

^a^Susceptible reference strain.

**Figure 2:**
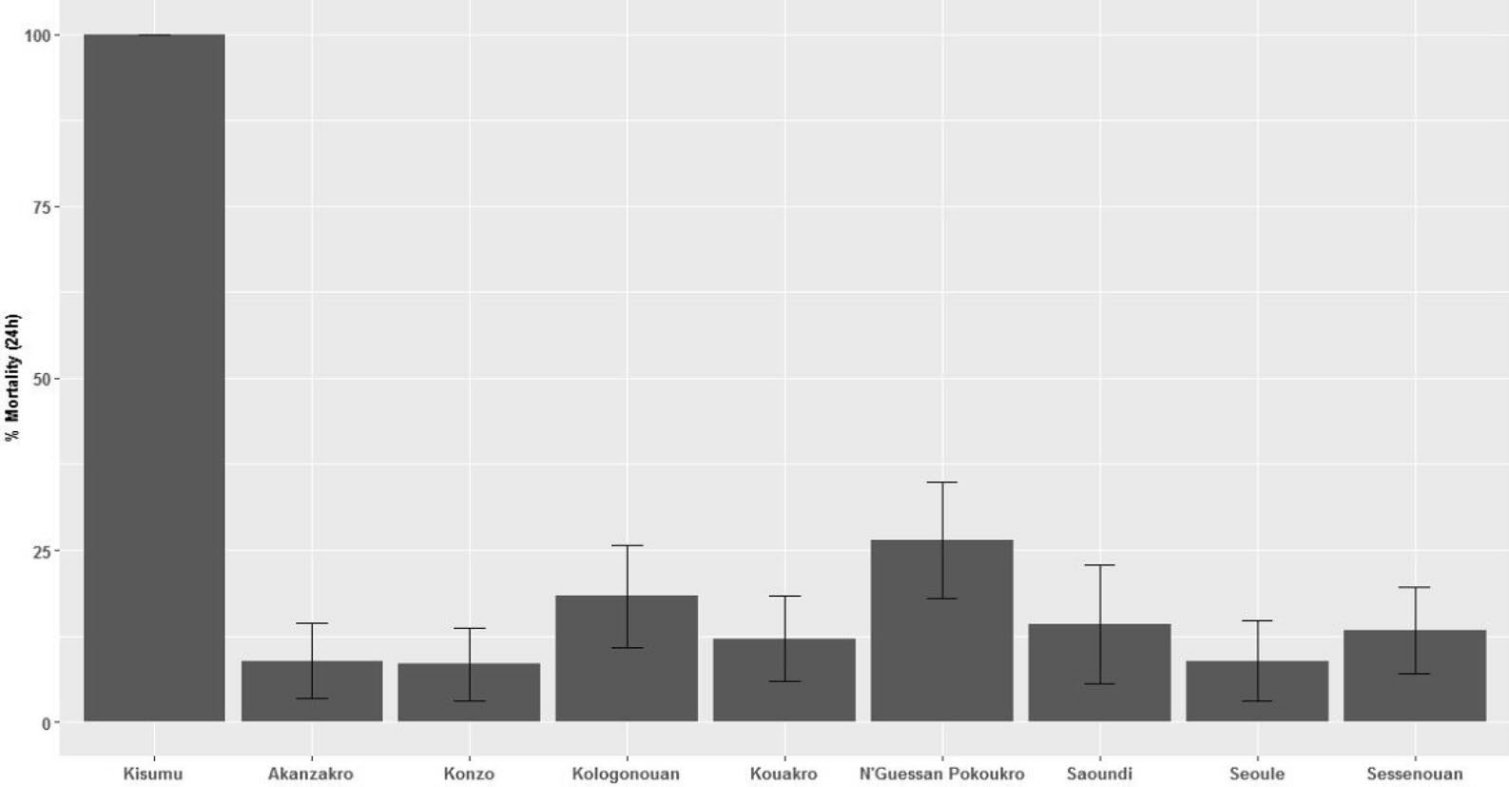
Percentage mortality of susceptible Kisumu and resistant *An. gambiae* s.l. exposed to LLIN material in WHO cone bioassays. Error bars indicate 95% Cis.

Exposure to a pyrethroid-only LLIN (PermaNet 2.0) killed 100% of the susceptible *An. gambiae* s.l. Kisumu strain but fewer than 30% *An. gambiae* s.l. mosquitoes from any study village (Fig. 2). Though the correlation between net-induced mortality and resistance intensity to deltamethrin was not significant (ρ = 0.41, n = 8, P = 0.32), the generally poor performance of the pyrethroid-only LLIN tested is consistent with the very high pyrethroid resistance in the villages. Mortality rates of mosquitoes exposed to LLIN material differed significantly between villages (Table 3).

**Table 3:** Generalised linear model testing the effects of village on net induced mortality.

	Village (arm)		
	Wald χ^2^	df	P
PermaNet 2.0	20.87	7	0.004

### Species identification and target-site resistance

Overall, 975 randomly selected *An. gambiae* s.l., which comprised of unexposed and pyrethroid bioassay survivors, were identified to species by PCR. A subset of these mosquitoes were screened for common resistance-linked *kdr* mutations in the voltage-gated sodium channel. The predominant malaria vector species in seven of the villages was *An. gambiae* (84–98%) with a single village (Kouakro) in which *An. coluzzii* and *An. gambiae* were found in comparable proportions (50%) (Table 4).

**Table 4:** Species composition in the study villages.

Study villages	*An. gambiae* (N)	*An. coluzzii* (N)	Hybrids (N)
Akanzakro	117	0	2
Kologonouan	86	0	0
Konzo	99	2	0
Kouakro	53	53	0
N’Guessan Pokoukro	160	12	1
Saoundi	116	2	0
Seoule	99	1	1
Sessenouan	158	12	1

N: number of *An. gambiae* s.l. mosquitoes identified to species by SINE-PCR.

The 1014S mutation was not detected in any of the 367 mosquito samples screened. The 1014F mutation was found at very high frequency (> 0.9) whereas the 1575Y allele was present at low frequency (< 0.15) in mosquito populations across villages. Allele frequencies of the 1014F mutation did not differ among villages (χ^2^_7_ = 12.2, P = 0.59) (Table 5). Likewise, allele frequencies of the 1575Y mutation were very similar across villages (χ^2^_7_ = 1.1, P = 0.99) (Table 5). The frequency of 1575Y also did not differ between unexposed mosquitoes and bioassay survivors (χ^2^_1_ = 0.05, P = 0.82). In each village, neither locus showed significant deviation from Hardy–Weinberg equilibrium (Table 5).

**Table 5:** Frequencies of 1014F and 1575Y *kdr* alleles in *An. gambiae* from study villages.

Study villages	N tested	L1014F					N1575Y				
		LL	LF	FF	F (1014F)	P value	NN	NY	YY	F (1575Y)	P value
Akanzakro	47	0	0	47	1	0.59	36	11	0	0.12	0.32
Kologonouan	46	0	1	45	0.99		38	6	2	0.11	
Konzo	48	0	4	44	0.96		35	13	0	0.14	
Kouakro	45	0	9	36	0.90		36	9	0	0.10	
N'Guessan Pokoukro	47	0	5	42	0.95		37	10	0	0.11	
Saoundi	41	1	4	36	0.93		34	6	1	0.11	
Seoule	40	1	1	38	0.96		31	8	1	0.13	
Sessenouan	53	0	0	53	1		43	9	1	0.10	

*N* number of samples, *L* leucine, *F* phenylalanine, *N* asparagine, *Y* tyrosine.

P values are from χ^2^-squared tests.

**Figure 3:**
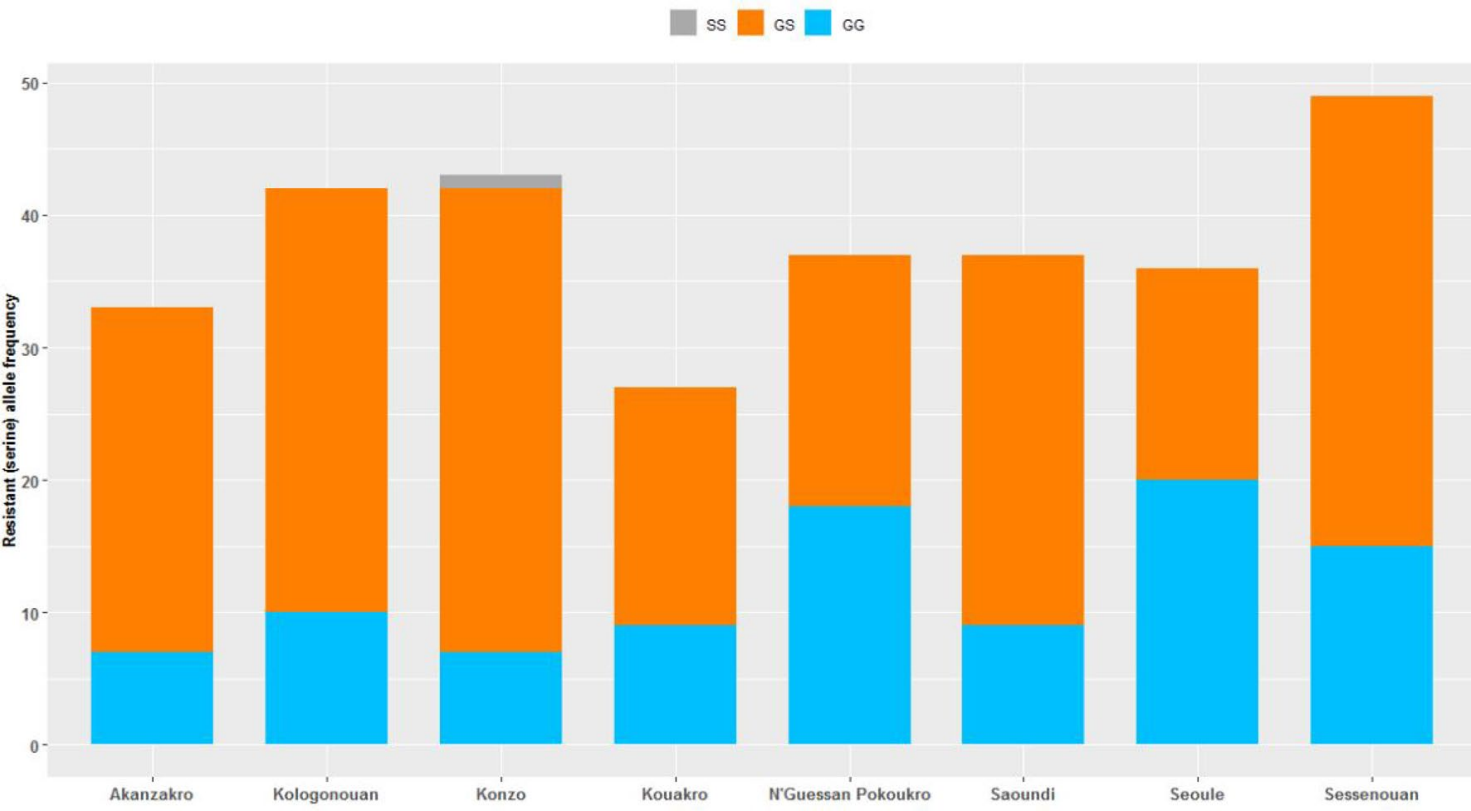
Genotypic frequencies of the *Ace-1* G119S mutation in *An. gambiae* mosquitoes. *GG* homozygote wild type; *GS* heterozygote resistant, *SS* homozygote resistant.

There was significant variation in allelic frequency of the G119S polymorphism across villages (22 to 43%; χ^2^_7_ = 22.75, P = 0.002), which essentially reflected variation in heterozygote vs susceptible homozygotes because resistant homozygotes were extremely rare (Fig. 3). Analysis of the qPCR dye balance ratio in heterozygotes, which can indicate variation in the relative number of duplicated serine alleles, showed no significant variation among villages in serine/glycine ratios (F_1,7_ = 0.94, P = 0.47), suggesting a similar copy number profile of serine alleles. There was no evidence that the frequency of G119S differed between *An. coluzzii* and *An. gambiae* in the mixed-species village of Kouakro (χ^2^_1_ = 1.2, P = 0.27).

### Genome-wide transcription analysis

Whole genome microarray experiments were conducted to identify candidate genes potentially involved in insecticide resistance in the dominant species *An. gambiae* collected from two of the study villages (N’Guessan Pokoukro and Sessenouan), in comparison with two susceptible strains, using a strict criterion for significance based on replicated fold change and multiple-testing corrected P-value thresholds.

**Figure 4:**
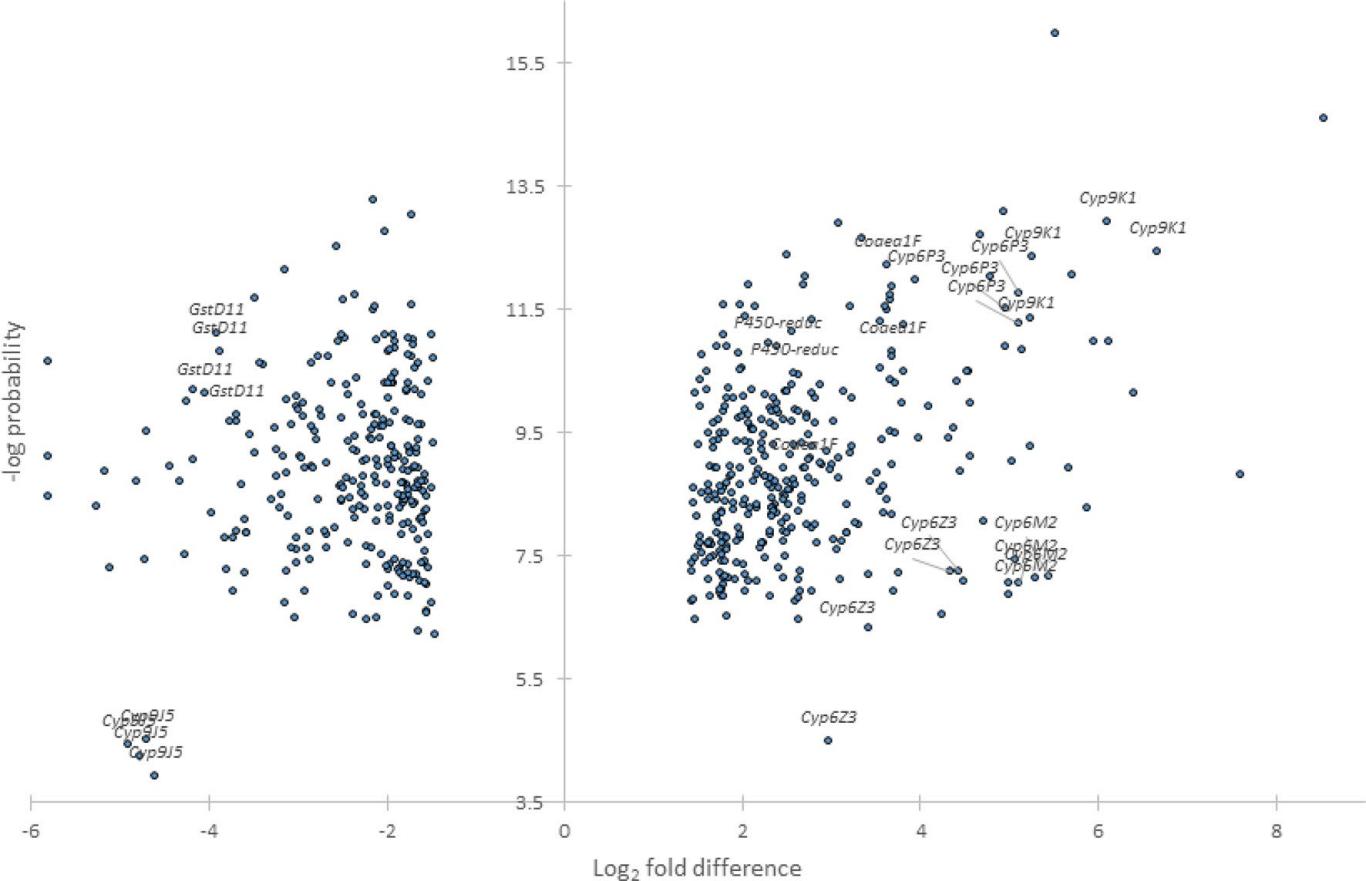
Differentially expressed probes in *An. gambiae* s.s. from two villages compared to two susceptible lab colonies. Average log_2_-transformed fold-differences are plotted against average negative log probabilities. Probes from genes chosen for qPCR validation are labelled.

Out of a total of 14,914 probes screened, 616 corresponding to 525 genes were significant according to the above filtering criteria (Fig. 4, Table S2A). Of the 267 genes (with 340 transcripts) over-expressed in all comparisons, we focused on those with known or putative links to detoxification or resistance more broadly, which comprised of 18 genes, including 11 cytochrome P450s, 3 glutathione S-transferases (GSTs), 2 carboxylesterases, an alcohol dehydrogenase, and peroxidase, a redox gene and transporters and cuticular genes (Table S2A). The three detoxification genes within the top 20 most over-expressed genes were cytochrome P450s (Table S2B) of which *Cyp6P3* and *Cyp9K1* exhibited > tenfold change and *Cyp6M2* with ≥ eightfold-change, but more variability across comparisons, relative to susceptible lab strains (Table S2B). Other highly over-expressed genes (within top 20) lack current description or have no putative link to insecticide resistance, based on current knowledge, such as the most highly expressed gene (h + transporting atp synthase subunit: fold change > 60). It is interesting to note that one of the two overexpressed esterases is the target site gene *Ace-1* with average overexpression of almost threefold, consistent with the expected presence of duplicated resistance alleles.

### Quantitative RT-PCR expression of candidate genes in selected villages

Candidate genes chosen for further analysis using qRT-PCR included the most over-expressed detoxification genes (*Cyp6P3*, *Cyp9K1*, and *Cyp6M2*), the most overexpressed esterase *COEA1F*, and the redox partner gene cytochrome P450 reductase. A further P450, *Cyp6Z3*, was chosen because it was significant in 3 out of 4 comparisons and we wished to examine whether the stringency of our filtering might be excluding potential valid detoxification candidates. The validation also included two under-expressed genes; one meeting the significant threshold across all comparisons (*GSTD11*) and one that was strongly underexpressed in one population (*Cyp9J5*), providing additional variation for qPCR vs microarray validation.

There was good agreement between qPCR and microarray estimates of gene expression (r^2^ = 0.73, P = 0.001) (Fig. S1). Fold change was generally higher in microarray results except for *Cyp6P3* and *Cyp9K1*, which showed higher expression in qPCR analysis.

**Figure 5:**
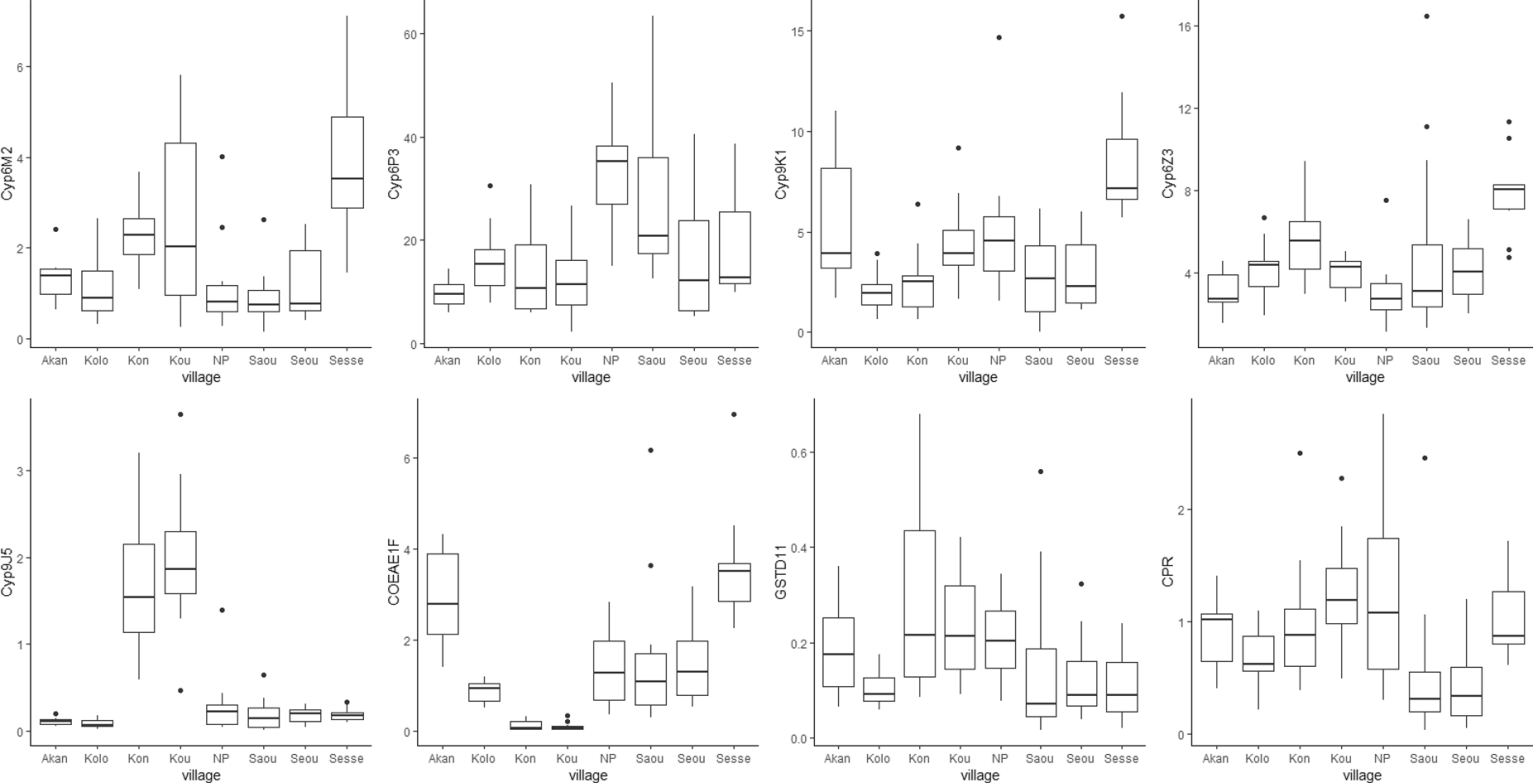
Box-whisker plots show mean fold difference in expression of candidate genes (relative to susceptible colony samples) across villages. The boxes represent the 25% and 75% quartiles and the whiskers indicate 5–95% quartile ranges. The horizontal line within each box represents the mean fold difference in gene expression, and the dots denote outliers.

The expression levels of the eight chosen candidate genes were assessed for variation across the eight villages. There was significant variation in the level of expression of all genes among villages (Kruskal Wallis tests, maximum P < 0.01) (Fig. 5 & Table S4A). The highest general level of expression was for *Cyp6P3*; with a particular peak in the N’Guessan Pokoukro village (33-fold change) but much lower levels in some other villages. Interestingly, there was a significant correlation between fold change in *Cyp6P3* and the intensity of resistance to deltamethrin (r^2^ = 0.78, P = 0.023) (Fig. S2). Expression level of all screened genes did not differ between unexposed mosquitoes and those that survived exposures to deltamethrin and cyfluthrin (Fig. S3, Table S4B).

## Discussion

Insecticide resistance in African malaria vectors is one of the major challenges facing malaria control programmes. A better understanding of the prevalence, intensity and mechanisms of resistance could inform the development of resistance management strategies. Results from the present study, the first of its kind on *An. gambiae s.s*. from Côte d’Ivoire, demonstrate phenotypic variation at a small spatial scale likely underpinned by variation in resistance mechanisms, notably P450 expression level and variation in *Ace-1* genotypic frequencies.

### Phenotypic resistance

High prevalence of resistance was evident for all insecticides tested, with the exception of pirimiphos methyl which was tested at a higher than diagnostic dose. These results are consistent with findings from previous studies conducted in the same area^21,32^. Multiple insecticide resistance has been previously documented in *An. coluzzii* from the southern part of the country (Tiassale)^10,21^. This observation is of significant concern for vector control, as resistance to non-pyrethroids limits the options for pyrethroid resistance management.

The intensity of deltamethrin resistance detected in the present study is among the highest reported to date in *Anopheles* mosquitoes. While quantitative measure of resistance enables detection of potential changes in resistance level in mosquito populations^33^, intensity level associated with operational control failure has yet to be defined. Nevertheless, the poor performance of LLINs in WHO cone assays against the local *Anopheles* mosquitoes is consistent with the high resistance intensity recorded and is suggestive of a potential loss of community protection from pyrethroid-only LLINs in this area.

### Resistance mechanisms

The molecular basis of the multiple insecticide resistance phenotype was investigated using microarray experiments performed on *An. gambiae* from two villages (one from each study arm). Analysis focused on overexpression of potential resistance-linked gene, but it should be noted that many genes of unknown function or no putative link to insecticide resistance were also significantly over-expressed in field mosquitoes compared to susceptible lab colonies. If this observation is reproducible, it could merit further investigation. Of the most highly overexpressed genes, *Cyp6P3* and *Cy6PM2* have been implicated repeatedly in pyrethroid resistance and also in resistance to carbamates in *An. gambiae* and/or *An. coluzzii*^10,34^ and are known to metabolize pyrethroids^35^. Overexpression of *Cyp9K1* has been linked to pyrethroid resistance in *An. gambiae* s.l. from Cameroon^12^, Benin^36^ and Bioko Island^28^, and has also recently been validated as a pyrethroid and pyriproxyfen metabolizer^28^. This is the first report of significant over-expression of *Cyp9K1* in Côte d’Ivoire, and the fold change in expression in mosquitoes from our study area is much higher than expression reported in previous studies^28,36^. The over-transcription of this set of P450s, coupled with the near fixation of *Vgsc* 1014F and the presence of the 1575Y mutations in the local malaria vectors, likely underpins the extreme resistance to pyrethroids and DDT in this part of Côte d’Ivoire. The carboxylesterase COEAE1F and the cytochrome P450 reductase (CPR) were among the significantly over-expressed detoxification candidates. Carboxylesterases can play a role in pyrethroid metabolism, for example when paired secondarily with P450s such as CYP6Z2^37^ (to which the candidate CYP6Z3 is extremely similar) and CPR is a redox partner for P450s and might also link with resistance^38^. These over-transcribed genes could have contributed to the high pyrethroid resistance observed. Although pyrethroid resistance in this population of mosquitoes is associated with both target site insensitivity and metabolic mechanisms, evidence from a recent study suggests that the latter resistance type is likely to account for the most extreme pyrethroid resistance intensity detected^11^. DDT resistance is often mediated by over-expression of Glutathione S transferases (GST) and *kdr*-based mechanisms. The absence of over-expressed GST indicates that the high DDT resistance might have been primarily due to 1014F and in some cases also 1575Y *kdr* mutations, perhaps coupled with overexpression of some genes less commonly associated with DDT resistance such as *Cyp6M2*^39^. The resistance intensifying mutation 1575Y was detected at relatively low frequency (< 15%) and found only in mosquitoes with the phenylalanine allele, confirming that this mutation only occurs on a 1014 haplotype background^17^. Originally identified in Burkina Faso, the 1575Y mutation is spreading across the continent and has been reported in West and Central Africa^13,40^. Understanding the key determinants behind the rapid increase in the prevalence of the 1575Y *kdr* allele could help slow or even stop the spread of this mutation. Further investigation is also needed to determine if the survival advantage associated with the co-occurrence of the 1575Y and 1014F^34^ mutations could negatively impact control efforts. The allelic frequency of this emerging gene should be closely monitored in areas where novel tools incorporating pyrethroids are deployed.

Carbamate resistance is primarily mediated by acetylcholinesterase insensitivity (G119S) and elevated expression of certain P450s^10^. The high survival to bendiocarb is consistent with the high frequency of *Ace-1* heterozygotes, which as shown by elevated *Ace-1* expression are likely present in higher copy numbers which raises carbamate resistance^10^. *Cyp6P3* was also over-expressed and has been shown to generate a moderately bendiocarb-resistant phenotype via transgenic expression and to metabolize bendiocarb, albeit with low catalytic efficiency. Indeed, susceptibility to bendiocarb in *An. gambiae* mosquitoes from Bioko has been reported despite over-expression of *Cyp6P3*^28^, and it may be that this is a mechanism of lesser importance. The role of *Cyp6M2*, which generates a much stronger resistance phenotype than *Cyp6P3* via transgenic expression but does not metabolise bendiocarb remains unclear, but it is certainly plausible that both combine with *Ace-1* copy number variation of resistant alleles to generate resistance phenotypes as observed in *An. coluzzii* from southern Côte d’Ivoire^10^.

Overall the resistance mechanisms detected in the study area are similar to those of *An. coluzzii* from southern Côte d’Ivoire^10^. These vector populations are from the same country and potentially exposed to the same insecticide selection pressure; mainly from the use of pyrethroid treated nets and insecticides for crop protection^2^. However, the elevated expression of the pyrethroid and pyriproxyfen metabolizing gene *Cyp9K1* in this study was not reported in the Tiassale mosquitoes. It could be that the frequency of this gene was low and undetectable at the time the Tiassale mosquito was characterised (in 2014) and might have increased only recently.

### Fine scale variation

The villages were all within 50 km radius away from the town of Bouaké and varied between a few km and a few tens of km apart. Yet, there was significant variation in both phenotypic data for all insecticides to which resistance was detected and in expression of all genes studied across villages. Monitoring of insecticide resistance in malaria vectors is often performed at large geographical scale. However, as seen in the present and previous studies^41,42^, variation in insecticide resistance can occur at small spatial scales. This result indicates the need to account for potential micro geographic variation during resistance surveys, rather than assuming broad-scale homogeneity for which single sites can act as reliable sentinels. Although wide-ranging phenotypic testing programmes incorporating fine-scale testing are unlikely to be realistic for most programmes, variation detected by molecular marker-based surveillance could aid in identifying sites of interest which could be prioritised for phenotypic testing. Interestingly, *Cyp6P3,* which showed the highest expression and high variation among villages correlated positively with resistance intensity suggesting a useful gene expression assay to predict resistance intensity.

## Conclusion

Results from this study are concerning given that anopheline mosquitoes from this part of Côte d’Ivoire have developed strong resistance to the main insecticides currently being used for malaria control. Metabolic genes that were found to be over-expressed in this study have previously been shown to metabolize some of the compounds being incorporated in new classes of bed nets. For example, a range of P450s, including those identified in the present study (*Cyp6P3*, *Cyp6M2* and *Cyp9K1*) metabolize pyriproxifen—an insect growth regulator deployed in nets to sterilise pyrethroid resistant mosquitoes^43^. This is consistent with the poor performance of Olyset Duo, a permethrin plus pyriproxyfen mixture LLIN in experimental huts in these areas of Côte d’Ivoire^44^ and in a randomised controlled trial in Burkina Faso^45^ where these pyriproxifen metabolizing genes were also found^11^. Use of PBO co-treated LLINs could be a more promising option in this area, given the apparent importance of P450 overexpression, though careful evaluation of efficacy and durability will be required.

The insecticide selected for use in the lethal house lure CRT is the pyrethroid beta-cyfluthrin^46^. This is because a previous study showed that the EaveTubes technology delivers an overwhelming dose of insecticide causing high levels of mortality of even resistant mosquitoes^46^. The data from the current study provides baseline information to track whether this additional use of pyrethroids on top of LLINs in the trial area will lead to changes in phenotypic resistance and associated molecular mechanisms.

## Methods

### Study area and collection of mosquitoes

This study was performed as part of a two-armed cluster randomized controlled trial (CRT) evaluating the impact of an intervention defined by the WHO Vector Control Advisory Group as a “lethal house lure”, which combines household screening (S) with a novel insecticide delivery system called In2Care EaveTubes (ET). The trial, which ran between May 2017 and May 2019 in central Côte d’Ivoire in the Gbèkè district, aimed to investigate whether the use of screening plus EaveTubes (SET) on top of universal coverage of LLINs (PermaNet 2.0), provides greater protection against malaria than LLINs alone. The design of the trial is described in Sternberg et al.^31^ and involves 40 villages, half assigned to SET plus LLINs, and the other half allocated to LLINs alone. The study area is a pre-forest zone with a humid tropical climate and covers an area of 9,136 km2 with a population of over one million people. Rice farming is the dominant form of subsistence agriculture and the presence of rice growing valleys across the region provides extensive breeding sites for anopheline mosquitoes. Malaria transmission is year-round with a peak during the rainy season (from May to October)^47,48^.

**Figure 6:**
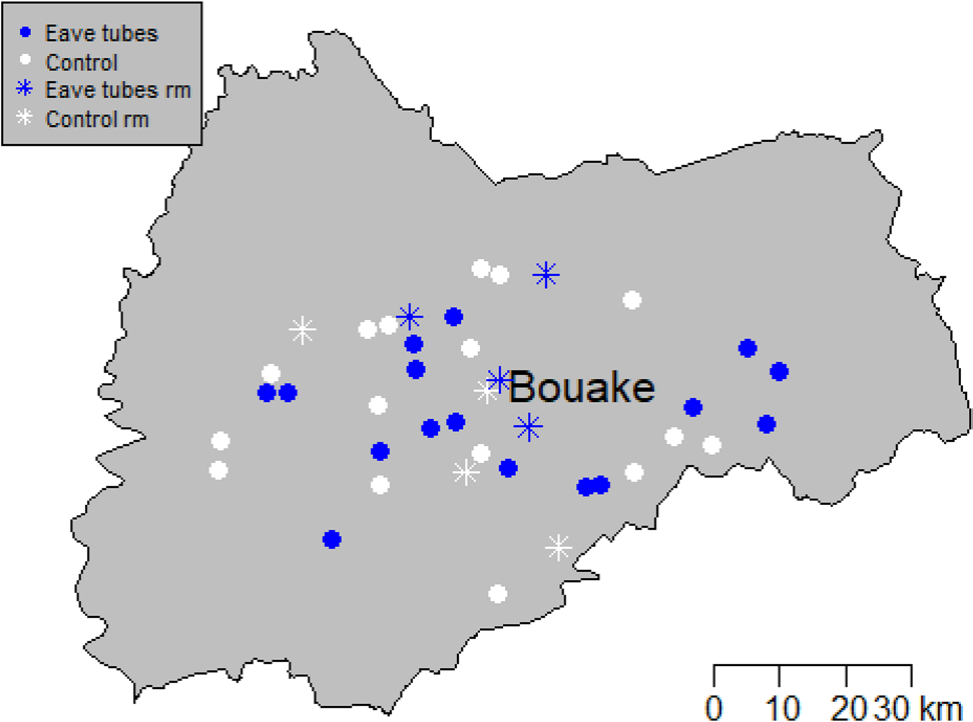
Map showing study villages involved in insecticide resistance monitoring (rm).

Eight study villages (four in each treatment arm) were selected for insecticide resistance monitoring, based on the availability of mosquito breeding sites for sampling (Fig. 6). A description of each sampling site is provided in Table 6. Mosquitoes were collected from each village using the dipping method from September 2016 to November 2016. Whenever possible, mosquito larvae were collected from at least two breeding sites spread out over the village, and collections from the same village were subsequently pooled. Larvae were transported to the insectary at the Institut Pierre Richet (IPR), fed on ground Tetramin fish food and reared to adulthood under ambient temperature. Emerging adult mosquitoes were kept in netted cages and maintained on 10% honey solution. All adult female mosquitoes were morphologically identified as *An. gambiae* s.l. using taxonomic keys.

**Table 6:** Location of study villages and description of mosquito breeding habitats.

Study village	Geographic coordinates		Arm	Type of breeding habitats
	Longitude	Latitude		
N'Guessan Pokoukro (NP)	7°56′N	5°20′W	Control (LLIN)	Water puddle
Kologonouan (Kolo)	7°66′N	5°17′W	Control (LLIN)	Water puddle
Konzo (Kon)	7°46′N	5°07′W	Control (LLIN)	Vegetable farm + rice field
Seoule Ahounzè (Seou)	7°76′N	5°42′W	Control (LLIN)	Rice field
Sessenouan (Sesse)	7°69′N	5°17′W	SET and LLIN	Vegetable farm + rice field
Kouakro (Koua)	7°83′N	5°08′W	SET and LLIN	Rice field + water puddle
Saoundi (Saou)	7°78′N	5°26′W	SET and LLIN	Rice field
Akazankro (Akan)	7°62′N	5°09′W	SET and LLIN	Vegetable farm + rice field

*SET* Screening plus EaveTubes, *LLIN* long-lasting insecticidal net.

### Insecticide susceptibility assays

To assess the prevalence of resistance in *Anopheles* mosquitoes from the CRT area (central Côte d’Ivoire), WHO susceptibility tests were performed between September and November 2016 using adult *An. gambiae* s.l. mosquitoes emerged from larvae collected in eight selected CRT villages. The pyrethroid insecticide, beta-cyfluthrin is the active deployed in the EaveTubes^46^ whereas deltamethrin is the insecticide in the LLIN (PermaNet 2.0). Bioassays were conducted using papers treated with diagnostic concentration of these two insecticides: 0.05% deltamethrin and 0.15% cyfluthrin. Additionally, susceptibility tests using paper impregnated with 4% DDT, 0.1% bendiocarb and 1% pirimiphos methyl were performed to assess the level of resistance to all four classes of WHO approved neurotoxic insecticides. The mosquitoes tested were 2–3 day-old adult female mosquitoes, emerged from larvae collected from study villages and reared in the insectary at IPR. Approximately 100 mosquitoes, in batch of 25, were exposed for 1 h to insecticide-treated papers, and mortality was recorded 24 h later. The same number of mosquitoes were exposed to untreated papers and served as control. Mosquitoes that survived exposure to either of the pyrethroids were monitored for an additional 24 h, after which the survivors were preserved in RNA later for subsequent molecular testing.

### Resistance intensity assays

To determine the intensity of resistance to pyrethroids in the local *Anopheles* mosquitoes, adapted CDC bottle assays were performed. Since both interventions (LLIN and EaveTubes) are treated with the same type of pyrethroids (pyrethroid type II), the intensity of pyrethroid resistance was determined using pyrethroid from one of these interventions. Bottles were coated with a range of deltamethrin concentrations (7.81 µg/mL to 1000 µg/mL), producing a range of mortality rates between 0 and 100% in mosquitoes from the study villages. Each bioassay included a control bottle treated with only acetone. The susceptible *An. gambiae* Kisumu strain (SS) served as reference and was tested against dosage range 0.001 µg/mL-0.5 µg/mL. Two to three days old adult female mosquitoes were exposed for 1 h at each concentration in four replicates of 25.

### WHO cone bioassay

To determine the impact of resistance on susceptibility to the bed nets (PermaNet 2.0) deployed in the study area, standard cone bioassays were performed according to WHO procedures using adult female mosquitoes emerged from larvae collected from the eight study villages and the susceptible Kisumu strain. Approximately 60 mosquitoes were exposed to a netting sample for 3 min and the mortality rate was determined 24 h later. Control mosquitoes (~ 60) were exposed to an untreated net and served as control.

### Species identification and target site resistance mechanisms

To type mosquitoes to species and identify target site resistance mechanisms in *Anopheles* mosquitoes from study villages, genomic DNA was extracted from a pair of legs taken from field mosquitoes that survived exposure to deltamethrin and cyfluthrin in WHO cylinder assays, and from a subset of unexposed female mosquitoes. The legs were boiled in 20µL of buffer solution for 90 min at 95 °C. Members of the *An. gambiae* complex were identified to species using SINE-PCR^49^.

TaqMan PCR assays were used to screen mosquito samples for mutations in the voltage gated sodium channel, including the 1014S, 1014F and 1575Y^17,50^, and for the *Ace-1* G119S^51^ resistance mutation in acetylcholinesterase. Heterozygotes for *An. gambiae* and *An. coluzzii* are all expected to include duplications in some combination of (1) G and S alleles are paired on a single chromosome—a heterogeneous duplication (2), an unduplicated G allele, and (3) a multicopy S allele^52^. Variation in composition of G and duplicated S alleles can be detected quantitatively as a difference in dye balance in heterozygotes in TaqMan qPCR^53^.

### Whole genome microarray

A genome-wide transcription profiling was performed to identify genes differentially expressed in mosquitoes from two CRT villages (one from each study arm) relative to susceptible lab strains. All of the villages involved in the CRT were at least 2 km apart; however, to capture the whole range of over/under expressed genes in mosquitoes from the study area, two villages much further away from each other were selected for microarray analysis. Mosquitoes used in microarray studies were confirmed as *An. gambiae* using SINE-PCR.

Gene expression profiles of unexposed, female *An. gambiae* mosquitoes from one control village (N’Guessan Pokoukro) and the survivors of deltamethrin exposure from one intervention village (Sessenouan) were compared to those of two susceptible lab strains, *An. gambiae* Kisumu and *An. gambiae* Ngousso, using an interwoven loop design (Fig. S4). Inclusion of survivors from one village and unexposed from another, with the highest prevalence of pyrethroid resistance maximised chances of identifying resistance-associated candidate genes, whilst ensuring that overexpression induced primarily by exposure (i.e. gene induction) was precluded. Field-collected mosquitoes included in the microarrays analysis were solely the most predominant species, *An. gambiae*. Significant differential expression between field mosquitoes from the two villages and the two insecticide susceptible lab strains was identified using a filtering approach. This was based on a P < 0.05 (after Bonferroni correction), a fold change in expression > 2 or < -2 and directionality i.e. the same direction of differential expression (upregulated or down-regulated) in the 4 comparisons (N’guessan Pokoukro vs Kisumu, N’guessan Pokoukro vs Ngousso, Sessenouan vs Kisumu, Sessenouan vs Ngousso). Total RNA was extracted from batches of ten female *An. gambiae* mosquitoes using a PicoPure RNA isolation kit (Thermo Fisher Scientific) according to the manufacturer’s protocol. Total RNA extracted from mosquitoes was treated using DNase (RNase free DNase set, Qiagen Hilden Germany). Before further use, the concentration and quality of the extracted RNA were evaluated using a NanoDrop spectrophotometer (Thermo Fisher Scientific) and a 2100 Bioanalyzer (Agilent Technologies). Four biological replicate extractions of total RNA for each mosquito population or colony were amplified and labelled using the Low Input Quick Amp Labeling Kit (Agilent Technologies). The Agilent Agam15k array was used for dual-color hybridizations (N’guessan Pokoukro vs Kisumu, N’guessan Pokoukro vs Ngoussou, Sessenouan vs Kisumu, Sessenouan vs Ngoussou)^39^. The labelled samples were hybridized using a Gene Expression Hybridization Kit (Agilent Technologies). Washing, scanning and feature extraction were performed according to the manufacturer’s recommendations. The design of the microarray experiment was optimized through comparison of the above strains across four microarray slides.

### Quantitative reverse transcriptase PCR for candidate gene expression in field mosquitoes

The expression of a subset of genes from microarray known to play a role in insecticide resistance in *An. gambiae* mosquitoes was taken forward for validation and measurement in field mosquitoes from the eight villages using reverse-transcription quantitative PCR (RT-qPCR). For each village, the expression for each gene of interest was measured in three cohorts of mosquitoes: non-exposed, deltamethrin and cyfluthrin survivors. Prior to qPCR experiments, RNA was extracted from field mosquitoes and quantified using the Nanodrop spectrophometer. cDNA was subsequently synthesized from 11 g of RNA using oligo(dT) 20 (50 μM) and SuperScript III (200U) (Invitrogen) and purified through a DNA-binding column (Qiagen). Three pairs of primers of each target gene were designed using Primer-BLAST tool (NCBI: https://www.ncbi.nhi.gov/tools/primers-blat/). The primer pair with the highest efficiency value (~ 100%), determined by running standard qPCR using serial dilution of a single cDNA sample, was selected for subsequent qPCR (details of the primers are given in Table S3). For each qPCR reaction, four biological replicates of each treatment group and two technical replicates were used. QPCR was performed using an Agilent Mx3005P QPCR System and the cycling condition was as follow: 95 °C for 3 min, 40 cycles of 95 °C for 10 s and 60 °C for 10 s. Expression of the genes was normalized using references genes (Ribosomal S7 and Elongation Factor).

### Statistical analysis

Mosquito mortality rates were compared using generalized linear models with a binary link function in SPSS v23. WHO assessments of mortality rates are: less than 90% indicates resistance; higher than 98% indicates susceptibility: between 90 and 98% requires further testing to confirm resistance status^54^. The intensity of resistance (Resistance Ratio, or RR50) was estimated using the R statistical software version 2.15.0 to compare the LD50 of the wild population relative to that of the susceptible lab strain. The variation in bioassay mortality rates of *An. gambiae* mosquitoes between villages was tested using a generalised linear model (GLM). The Spearman test was used to test the correlation between resistance intensity to deltamethrin and bioassay mortality rates. The frequencies of target site resistance mutations in field anopheline populations were compared between study villages using a χ^2^-square test with Yates continuity correction. Concordance with Hardy–Weinberg equilibrium was assessed for each resistance marker in each village using the permutation-based probability test in Genepop^55,56^, with Bonferroni correction applied for multiple testing.

A MAANOVA model was used to analyse microarray data using previously described custom R-scripts^39^. Differentially expressed genes (over/under expressed) were those with a fold change consistently greater than 2 or less than -2 across the four comparisons (N’guessan Pokoukro vs Kisumu, N’guessan Pokoukro vs Ngousso, Sessenouan vs Kisumu, Sessenouan vs Ngousso) and with a significant Bonferroni-corrected p value in all four comparisons.

Outliers were identified and excluded from the qPCR dataset prior to analysis. The ΔΔCt method incorporating PCR efficiency was used to compare expression of each target gene between field mosquitoes and the lab strain^57^. Significant difference in fold change between field samples and the reference lab colony was estimated using a t-test (P < 0.05). Kruskal Wallis test was used to compare the level of expression of candidate genes across the three groups of field mosquitoes (unexposed group and mosquitoes surviving exposure to the two different pyrethroids in WHO cylinder assays).

## Supplementary information

Supplementary Figures

Supplementary Tables

## Data Availability

All data generated or analysed during this study are included in this published article (and its Supplementary Information files).

## References

[1] Bhatt S (2015). The effect of malaria control on *Plasmodium falciparum* in Africa between 2000 and 2015. Nature.

[2] Reid MC, McKenzie FE (2016). The contribution of agricultural insecticide use to increasing insecticide resistance in African malaria vectors. Malar. J..

[3] Czeher C, Labbo R, Arzika I, Duchemin J-B (2008). Evidence of increasing Leu-Phe knockdown resistance mutation in *Anopheles gambiae* from Niger following a nationwide long-lasting insecticide-treated nets implementation. Malar. J..

[4] Ngufor C (2016). Efficacy of the Olyset Duo net against insecticide-resistant mosquito vectors of malaria. Sci. Transl. Med..

[5] N’Guessan R (2016). A chlorfenapyr mixture net interceptor G2 Shows high efficacy and wash durability against resistant mosquitoes in West Africa. PLoS ONE.

[6] Protopopoff N (2018). Effectiveness of a long-lasting piperonyl butoxide-treated insecticidal net and indoor residual spray interventions, separately and together, against malaria transmitted by pyrethroid-resistant mosquitoes: a cluster, randomised controlled, two-by-two fact. The Lancet.

[7] Oxborough RM (2015). A new class of insecticide for malaria vector control: evaluation of mosquito nets treated singly with indoxacarb (oxadiazine) or with a pyrethroid mixture against *Anopheles gambiae* and *Culex quinquefasciatus*. Malar. J..

[8] Ngufor C (2017). Which intervention is better for malaria vector control: insecticide mixture long-lasting insecticidal nets or standard pyrethroid nets combined with indoor residual spraying?. Malar. J..

[9] Ngufor C, Fongnikin A, Rowland M, N’Guessan R (2017). Indoor residual spraying with a mixture of clothianidin (a neonicotinoid insecticide) and deltamethrin provides improved control and long residual activity against pyrethroid resistant *Anopheles gambiae* sl in Southern Benin. PLoS ONE.

[10] Edi CV (2014). CYP6 P450 enzymes and *ACE-1* duplication produce extreme and multiple insecticide resistance in the malaria mosquito *Anopheles gambiae*. PLoS Genet..

[11] Toé KH, N’Falé S, Dabiré RK, Ranson H, Jones CM (2015). The recent escalation in strength of pyrethroid resistance in *Anopheles coluzzi* in West Africa is linked to increased expression of multiple gene families. BMC Genom..

[12] Antonio-Nkondjio C (2017). Review of the evolution of insecticide resistance in main malaria vectors in Cameroon from 1990 to 2017. Parasites Vectors.

[13] Lynd A (2018). Insecticide resistance in *Anopheles gambiae* from the northern Democratic Republic of Congo, with extreme knockdown resistance (*kdr*) mutation frequencies revealed by a new diagnostic assay. Malar. J..

[14] Govere J (2018). Insecticide resistance status of the malaria mosquitoes: *Anopheles gambiae* and *Anopheles funestus* in eastern and northern Uganda. Malar. J..

[15] Martinez-Torres D (1998). Molecular characterization of pyrethroid knockdown resistance (kdr) in the major malaria vector *Anopheles gambiae* s.s. Insect Mol. Biol..

[16] Ranson H (2000). Identification of a point mutation in the voltage-gated sodium channel gene of Kenyan *Anopheles gambiae* associated with resistance to DDT and pyrethroids. Insect Mol. Biol..

[17] Jones CM (2012). Footprints of positive selection associated with a mutation (N1575Y) in the voltage-gated sodium channel of *Anopheles gambiae*. Proc. Natl. Acad. Sci..

[18] Wang L (2015). A Mutation in the intracellular loop III / IV of mosquito sodium channel synergizes the effect of mutations in Helix IIS6 on pyrethroid resistance. Mol. Pharmacol..

[19] Djogbénou L (2007). Characterization of insensitive acetylcholinesterase (ace-1 R ) *in Anopheles gambiae* (Diptera: Culicidae): resistance levels and dominance. J. Med. Entomol..

[20] Essandoh J, Yawson AE, Weetman D (2013). Acetylcholinesterase (*Ace-1*) target site mutation 119S is strongly diagnostic of carbamate and organophosphate resistance in *Anopheles gambiae* s.s. and *Anopheles coluzzii* across southern Ghana. Malaria Journal.

[21] Edi CVA, Koudou BG, Jones CM, Weetman D, Ranson H (2012). Multiple-insecticide resistance in *Anopheles gambiae* mosquitoes, Southern Côte d’Ivoire. Emerg. Infect. Dis..

[22] Djogbénou L, Noel V, Agnew P (2010). Costs of insensitive acetylcholinesterase insecticide resistance for the malaria vector *Anopheles gambiae* homozygous for the G119S mutation. Malar. J..

[23] Djogbénou L (2009). *Ace-1* duplication in *Anopheles gambiae*: a challenge for malaria control. Malar. J..

[24] Duangkaew P (2011). Characterization of mosquito cyp6p7 and cyp6aa3: differences in substrate preference and kinetic properties. Arch. Insect Biochem. Physiol..

[25] Riveron JM (2012). Directionally selected cytochrome P450 alleles are driving the spread of pyrethroid resistance in the major malaria vector *Anopheles funestus*. Proc. Natl. Acad. Sci. USA.

[26] Riveron JM (2014). The highly polymorphic *CYP6M7* cytochrome P450 gene partners with the directionally selected *CYP6P9a* and *CYP6P9b* genes to expand the pyrethroid resistance front in the malaria vector *Anopheles funestus* in Africa. BMC Genom..

[27] Mitchell SN (2012). Identification and validation of a gene causing cross-resistance between insecticide classes in *Anopheles gambiae* from Ghana. Proc. Natl. Acad. Sci. USA.

[28] Vontas J (2018). Rapid selection of a pyrethroid metabolic enzyme CYP9K1 by operational malaria control activities. Proc. Natl. Acad. Sci. USA.

[29] Camara S (2018). Mapping insecticide resistance in *Anopheles gambiae* (s.l.) from Côte d’Ivoire. Parasites Vectors.

[30] Koffi AA (2012). Update on resistance status of *Anopheles gambiae* s.s. to conventional insecticides at a previous WHOPES field site "Yaokoffikro", 6 years after the political crisis in Côte d’Ivoire. Parasites & Vectors.

[31] Sternberg ED (2018). Evaluating the impact of screening plus eave tubes on malaria transmission compared to current best practice in central Côte d’Ivoire: a two armed cluster randomized controlled trial. BMC Public Health.

[32] Zoh DD (2018). The current insecticide resistance status of *Anopheles gambiae* (s.l.) (Culicidae) in rural and urban areas of Bouaké, Côte d’Ivoire. Parasites Vectors.

[33] Bagi J (2015). When a discriminating dose assay is not enough: measuring the intensity of insecticide resistance in malaria vectors. Malaria Journal.

[34] Donnelly MJ, Isaacs AT, Weetman D (2016). Identification, validation, and application of molecular diagnostics for insecticide resistance in malaria vectors. Trends Parasitol..

[35] Stevenson BJ (2011). Cytochrome P450 6M2 from the malaria vector *Anopheles gambiae* metabolizes pyrethroids: Sequential metabolism of deltamethrin revealed. Insect Biochem. Mol. Biol..

[36] Ngufor C (2015). Insecticide resistance profile of *Anopheles gambiae* from a phase II field station in Cové, southern Benin: implications for the evaluation of novel vector control products. Malar. J..

[37] Chandor-Proust A (2013). The central role of mosquito cytochrome P450 CYP6Zs in insecticide detoxification revealed by functional expression and structural mode. Biochem. J..

[38] Hemingway J (2006). *Anopheles gambiae* P450 reductase is highly expressed in oenocytes and in vivo knockdown increases permethrin. Insect Mol. Biol..

[39] Mitchell SN (2014). Metabolic and target-site mechanisms combine to confer strong DDT resistance in *Anopheles gambiae*. PLoS ONE.

[40] Edi AVC (2017). First detection of N1575Y mutation in pyrethroid resistant *Anopheles gambiae* in Southern Côte d’Ivoire. Wellcome Open Res..

[41] Deming R (2016). Spatial variation of insecticide resistance in the dengue vector *Aedes aegypti* presents unique vector control challenges. Parasites Vectors.

[42] Matowo NS (2017). Fine-scale spatial and temporal heterogeneities in insecticide resistance profiles of the malaria vector, *Anopheles arabiensis* in rural south-eastern Tanzania. Wellcome Open Res..

[43] Yunta C (2016). Pyriproxyfen is metabolized by P450s associated with pyrethroid resistance in *An. gambiae*. Insect Biochem. Mol. Biol..

[44] Koffi AA (2015). Efficacy of Olyset Duo, a permethrin and pyriproxyfen mixture net against wild pyrethroid-resistant *Anopheles gambiae* s.s. from Côte d’Ivoire: an experimental hut trial. Parasite.

[45] Tiono AB (2018). Efficacy of Olyset Duo, a bednet containing pyriproxyfen and permethrin, versus a permethrin-only net against clinical malaria in an area with highly pyrethroid-resistant vectors in rural Burkina Faso: a cluster-randomised controlled trial. Lancet.

[46] Oumbouke WA (2018). Screening and field performance of powder-formulated insecticides on eave tube inserts against pyrethroid resistant *Anopheles gambiae* s.l.: an investigation into ‘actives’ prior to a randomized controlled trial in Côte d’Ivoire. Malar. J..

[47] Diakité NR, Adja AM, Von Stamm T, Utzinger J, N’Goran EK (2010). Situation épidémiologique avant la mise en eau du barrage hydroagricole de cinq villages de Bouaké, Centre Côte-d’Ivoire. Bull. Soc. Pathol. Exotique.

[48] Diakité NR (2015). Spatial and temporal variation of malaria entomological parameters at the onset of a hydro-agricultural development in central Côte d’Ivoire. Malar. J..

[49] Santolamazza F (2008). Insertion polymorphisms of SINE200 retrotransposons within speciation islands of *Anopheles gambiae* molecular forms. Malar. J..

[50] Bass C (2007). Detection of knockdown resistance (*kdr*) mutations in *Anopheles gambiae*: a comparison of two new high-throughput assays with existing methods. Malaria Journal.

[51] Bass C (2010). The Vector Population Monitoring Tool (VPMT): high-throughput DNA-based diagnostics for the monitoring of mosquito vector populations. Malar. Res. Treat..

[52] Weetman D, Djogbenou LS, Lucas E (2018). Copy number variation (CNV) and insecticide resistance in mosquitoes: evolving knowledge or an evolving problem?. Curr. Opin. Insect Sci..

[53] Djogbénou LS (2015). Estimation of allele-specific *Ace-1* duplication in insecticide-resistant *Anopheles* mosquitoes from West Africa. Malar. J..

[54] WHO (2013). Guidelines for Laboratory and Field Testing of Long-Lasting Insecticidal Mosquito Nets.

[55] Raymond M, Rousset F (1995). GENEPOP (Version 12): population genetics software for exact tests and ecumenicism. J. Hered..

[56] Rousset F (2008). GENEPOP’007: A complete re-implementation of the GENEPOP software for Windows and Linux. Mol. Ecol. Resour..

[57] Schmittgen TD, Livak KJ (2008). Analyzing real-time PCR data by the comparative CT method. Nat. Protoc..

