# Supplementary material for: Fine scale spatial investigation of multiple insecticide resistance and underlying target-site and metabolic mechanisms in Anopheles gambiae in central Côte d’Ivoire
Source: Sci Rep. 2020 Sep 15;10:15066. doi: 10.1038/s41598-020-71933-8 (PMC7493912; doi:10.1038/s41598-020-71933-8)
Supplement: Supplementary file 1 — Supplementary Figures [file 41598_2020_71933_MOESM1_ESM.docx]

**Fine scale spatial investigation of multiple insecticide resistance and underlying target-site and metabolic mechanisms in *Anopheles gambiae* in central Côte d’Ivoire**

Welbeck A. Oumbouke^1,2^*, Patricia Pignatelli^3^, Antoine M.G. Barreaux^4,5^, Innocent Z. Tia^2^, Alphonsine A. Koffi^2^, Ludovic P. Ahoua Alou^2^, Eleanore D. Sternberg^5^, Matthew B. Thomas^5^, David Weetman† ^3^ and Raphael N’Guessan† ^1,2^

**Supplementary Figures**


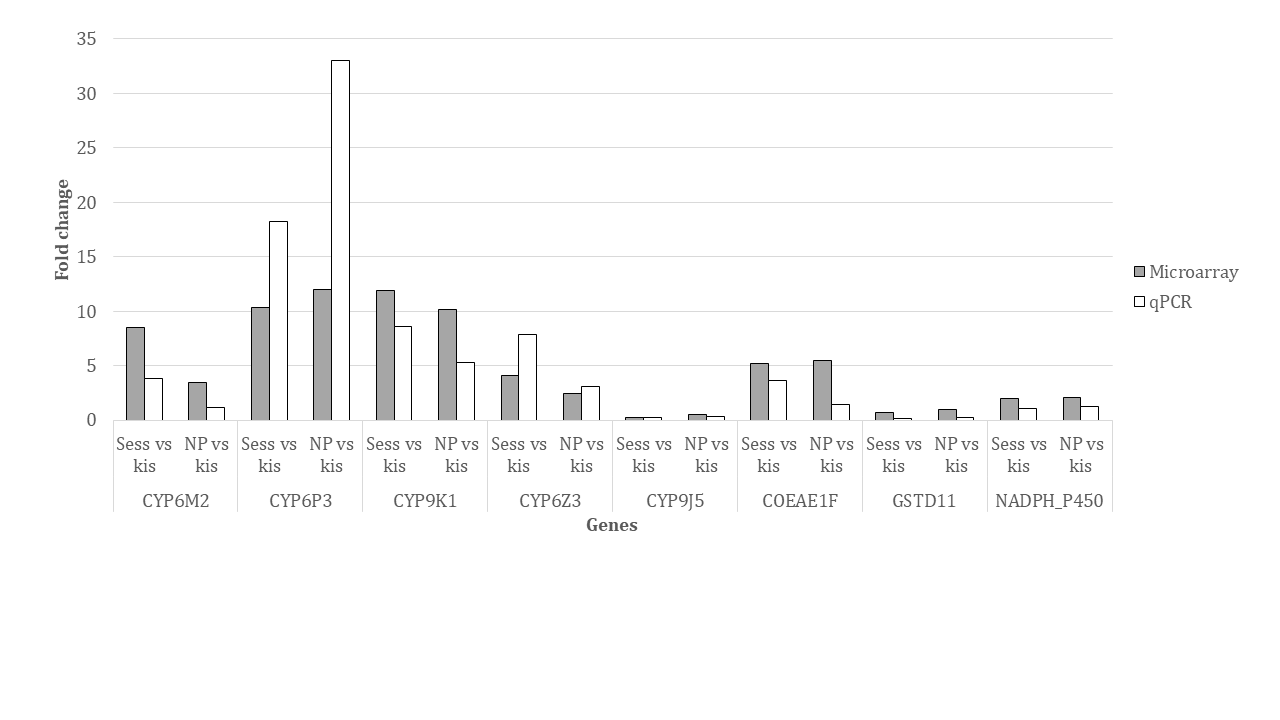


**Figure S1: Side-by-side fold change in gene expression measured by microarrays and qRT-PCR for selected candidate genes.** The overall correlation is r^2^= 0.73.

**
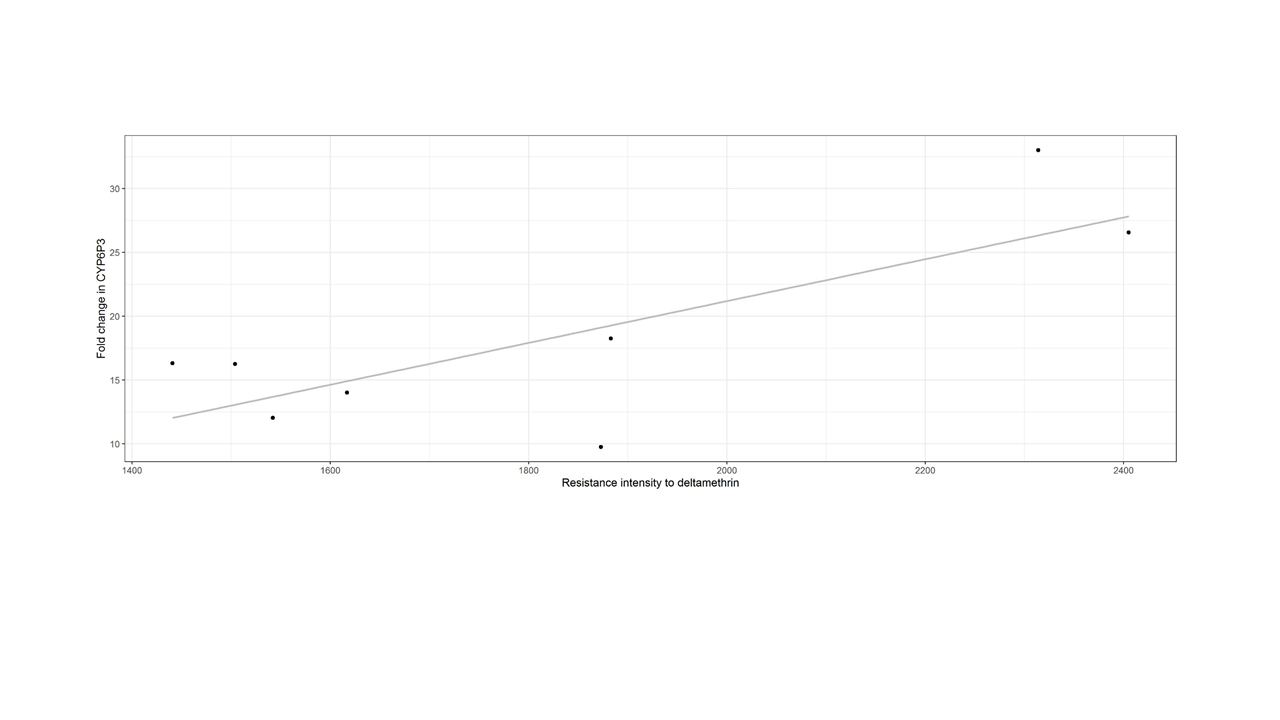
**

**Figure S2: Association between fold change in *Cyp6P3* and resistance intensity to deltamethrin**


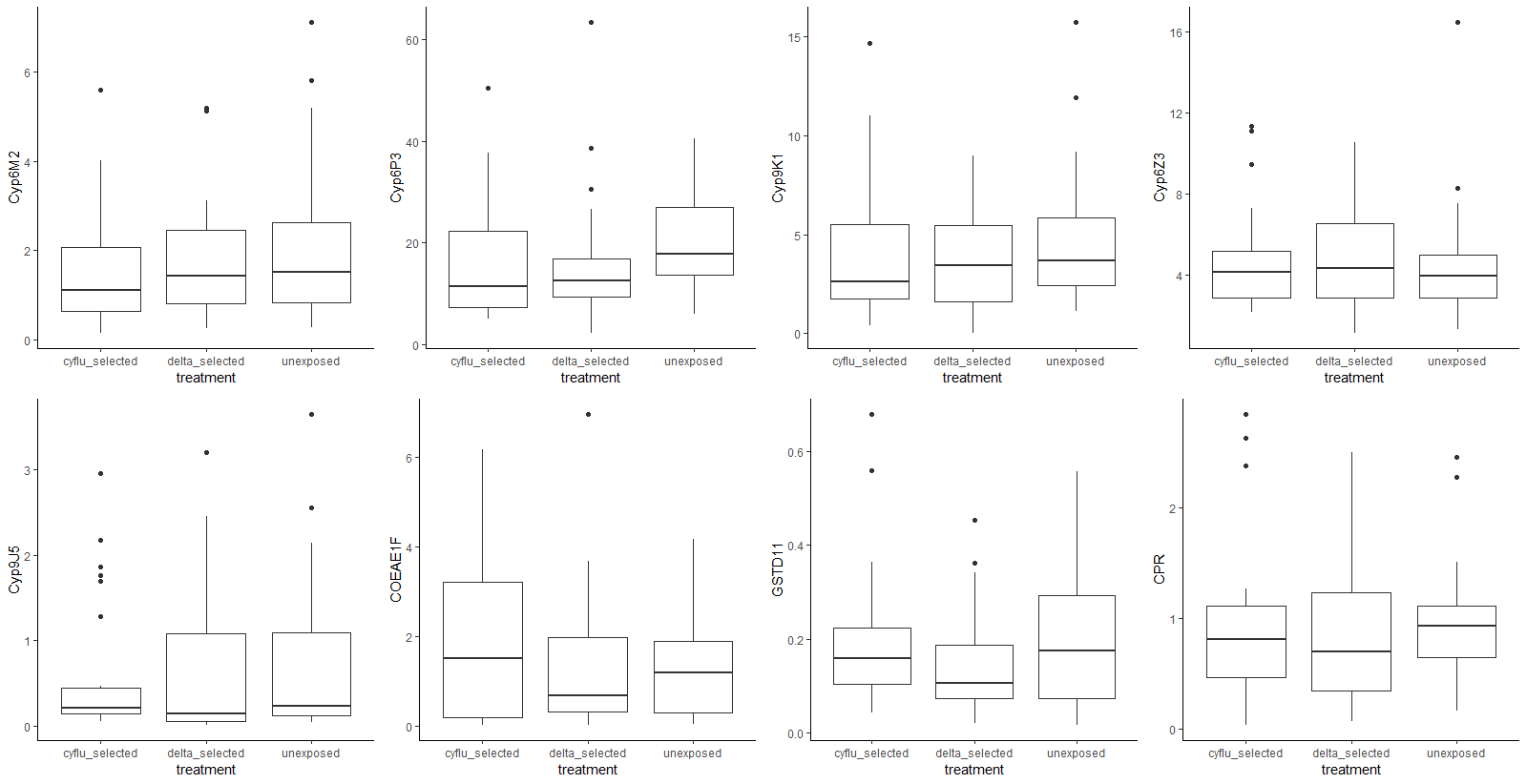


**Figure S3: Boxplots show mean fold change in expression of candidate genes across treatments.** The boxes represent the 25% and 75% quartiles and the whiskers indicate 5% - 95% quartile ranges. The horizontal line within each box represents the mean fold difference in gene expression and the dots denote outliers.


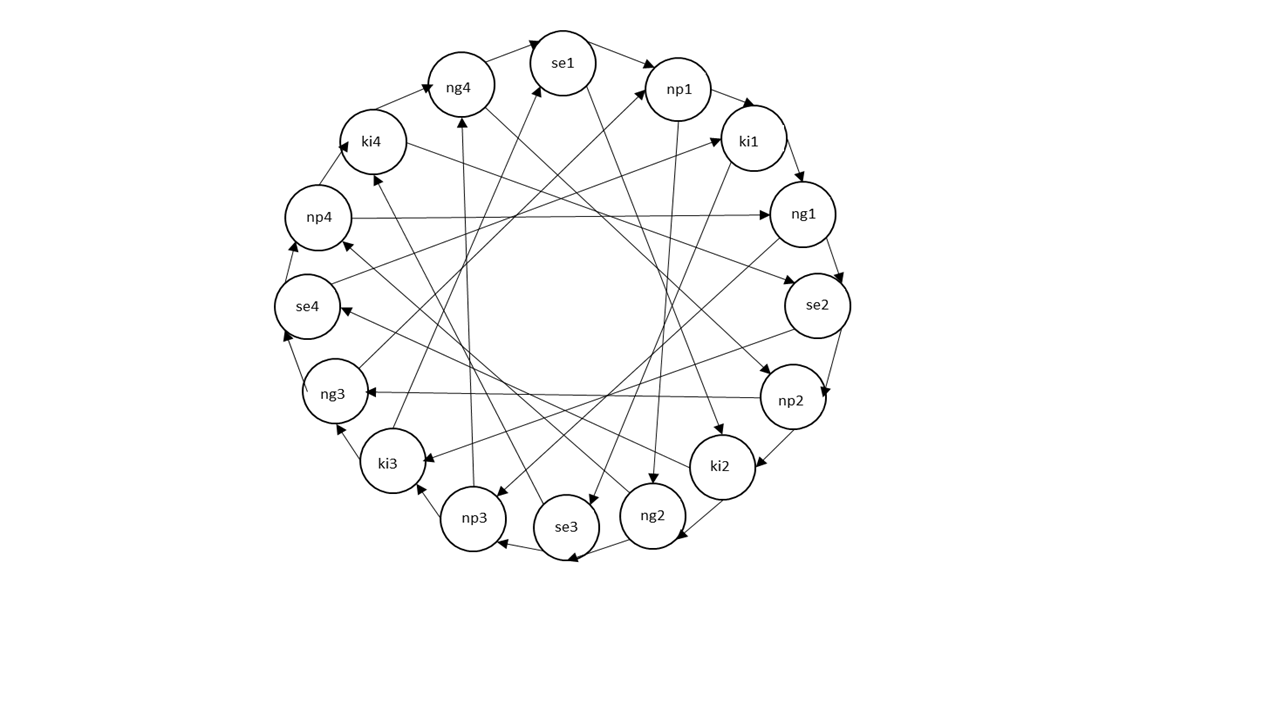


**Figure S4: Interwoven microarray loop design comparing field mosquito samples from two CRT villages (one control cluster: np=N’Guessan Pokoukro and one intervention cluster: se=Sessenouan) and two lab colonies (kis= *An. gambiae* Kisumu and ng= *An. gambiae* N’goussou).** Each circle represents mRNA extracted from a pool of 10 female *An. gambiae* s.s. Individuals microarrays are represented by arrows (32 in total). The direction of the arrows indicates dye labelling.
